# Educational climate perception by preclinical and clinical medical students in five Spanish medical schools

**DOI:** 10.5116/ijme.5557.25f9

**Published:** 2015-06-08

**Authors:** Jorge Pales, Arcadi Gual, Jesus Escaneroi, Inmaculada Tomás, Felipe Rodríguez de Castro, Marta Elorudy, Montserrat Virumbrales, Gerardo Rodríguez, Victor Arce

**Affiliations:** 1 School of Medicine, University of Barcelona, Spain; 2 School of Medicine, University of Zaragoza, Spain; 3 School of Medicine and Dentistry, University of Santiago de Compostela, Spain; 4 School of Health Sciences, University of Las Palmas de Gran Canaria, Spain; 5 School of Medicine and Health Sciences, International University of Catalonia, Spain

**Keywords:** Educational climate, undergraduate curriculum, DREEM

## Abstract

**Objectives:**

The purpose of this study was to investigate student's perceptions of Educational Climate (EC) in Spanish medical schools, comparing various aspects of EC between the 2^nd^ (preclinical) and the 4^th^ (clinical) years to detect strengths and weaknesses in the on-going curricular reform.

**Methods:**

This study utilized a cross-sectional design and employed the Spanish version of the "Dundee Ready Education Environment Measure" (DREEM). The survey involved 894 2^nd^ year students and 619 4^th^ year students from five Spanish medical schools.

**Results:**

The global average score of 2^nd^ year students from the five medical schools was found to be significantly higher (116.2±24.9, 58.2% of maximum score) than that observed in 4th year students (104.8±29.5, 52.4% of maximum score). When the results in each medical school were analysed separately, the scores obtained in the 2^nd^ year were almost always significantly higher than in the 4^th^ year for all medical schools, in both the global scales and the different subscales.

**Conclusions:**

The perception of the EC by 2^nd^ and 4^th^ year students from five Spanish medical schools is more positive than negative although it is significantly lower in the 4^th^ year. In both years, although more evident in the 4^th^ year, students point out the existence of several important "problematic educational areas" associated with the persistence of traditional curricula and teaching methodologies. Our findings of this study should lead medical schools to make a serious reflection and drive the implementation of the necessary changes required to improve teaching, especially during the clinical period.

## Introduction

The educational environment is a key element of student learning and a reflection of the quality of the curriculum. The perception of the educational environment may be designated as the educational climate (EC) and has been defined as "the soul and the spirit of the medical school environment and curriculum";.^1^ Therefore, the EC is considered to mean "everything that is happening in the classroom, in a department, in the medical school, or in the university".^1^ Different elements influence the EC; well defined learning outcomes, teacher's competencies, learning resources, learning and teaching methodologies, assessment methods, timetabling, student support, facilities, classrooms, group size and the atmosphere, amongst others.^2^ As well as influencing teacher behaviour and the behavioural development of students and vice-versa, the EC must be taken into consideration because of its implication in the achievement of corporate goals and the level of satisfaction obtained.^2^^-^^6^ Finally, EC has been considered as the expression, manifestation and measure of a curriculum and as a stimulus for change.^2^^,^^7^

One of the principal tools to measure the EC is the Dundee Ready Educational Environment Measure (DREEM), developed by Roff et al. in 1997.^8^ The questionnaire has been translated into different languages including Spanish,^9^^-^^10^ validated and widely used for studies in various countries across Europe, Asia, Africa, North America, South America, and the Middle East.^2^^,^^6^^,^^9^^-^^26^ These studies have achieved a number of goals, including the generation of a profile of the strengths and weaknesses of an institution or course that allows the creation of comparative analyses either within an institution or between institutions, which can then be applied as predictors of student performance.

In the context of Spanish Medical Schools, besides the pioneering studies of Escanero et al.^27^ in 2009, which were undertaken before implementation of the Bologna reform, there are only two partial studies comparing Spanish Medical Schools with Argentinian and Chilean counterparts.^21^^-^^23^ The most complete Spanish study to date, a psychometric validation of the Spanish version of the DREEM with application to Spanish dentistry students, was performed by Tomás et al. in 2013.^28^

Since 2009, in the context of the Bologna reform process, Spanish Medical Schools have been implementing new curricula. These new curricula are 6 years in duration with 360 ECTS (European Credit Transfer System). In spite of the declared objective of implementing an outcome-based curriculum, Spanish medical schools maintain very traditional and teacher-centred curricula with the conventional separation between the preclinical and clinical periods. Moreover, traditional methods of teaching, learning and assessment are still used. However, in some medical schools there are examples of innovation such as the introduction of problem-based learning strategies, the use of simulations for learning, the implementation of OSCES and an increase in the time devoted to clinical practice. Nonetheless, students from the 1st and 2nd years continue to have little contact with clinical aspects, which begins in the 3rd year and is completed in the 4th and successive years.

In order to gauge how these new curricula are developing, the Spanish Society for Medical Education (SEDEM) decided to undertake a cross-sectional study in different medical schools to analyse the perception of EC by 2nd and 4th year students to determine: a) whether the perception of the EC by medical students, is different between the 2nd and the 4th year, (i.e. basic versus clinical period); b) how the curricular change is developing, particularly after the transition between the preclinical and clinical periods, and c) the strengths and weaknesses in the new curriculum as they relate to the educational environment after 4 years of Bologna implementation. Although Spanish medical schools have very similar curriculum structures, they show some differences regarding their character (public or private), foundation (older and newer medical schools) and their size and number of students. Given these differences and in order to avoid establishing any ranking among them, the comparison between different medical schools per se was not a goal of this study. The emphasis of the study was placed on the comparison between preclinical and clinical years in each medical school. However, each individual medical school, upon viewing its own results, can be made aware of its particular situation.

## Methods

### Study design

SEDEM invited Spanish Medical Schools to enrol in this project and provided the required support to develop the project and facilitate data collection. A cross-sectional survey design was implemented. Students from the 2nd (preclinical) and 4th (clinical) years from the different Spanish medical schools were invited to participate in the study.

### Participants

Five schools decided to join the study (4 public and 1 private). This study was carried out with medical students from the 2nd and the 4th years of the following universities (in alphabetical order): Barcelona (UB), Internacional de Catalunya (UIC), Las Palmas de Gran Canaria (ULPGC), Santiago de Compostela (USC) and Zaragoza (UNIZAR). The University of Barcelona and SEDEM served as the coordinators of the project. For the purposes of presenting the results, the different schools were randomly designated as M1, M2, M3, M4 and M5. The study was approved by the CEIC (Ethical Research Committee) of the Clinic Hospital-Medical School-University of Barcelona.

### Instruments

The instrument used in this study was the Dundee Ready Educational Environment Measure (DREEM). The DREEM is a 50-statement, closed-question questionnaire developed by Roff et al^8^ to measure the learning environment of educational establishments. Each of the 50 items falls into one of five subscales/domains relating to different aspects of students' perception: perception of learning (LP); perception of teachers (TP); academic self-perception (AP); perception of atmosphere (AtmP), and social self-perception (SP). Each of the 50 statements is scored on a 5-point scale, expressing the degree of agreement with each statement (i.e. 4 strongly agree, 3 agree, 2 not sure, 1 disagree and 0 strongly disagree). For several items, a reverse coding is required (statements 4, 8, 9, 17, 25, 35, 39, 48 and 50). The DREEM scale provides results for each item, for each domain (the sum of the scores of the corresponding items) and for total EC score (the sum of the scores of each domain). The maximum possible scores for the different domains are: LP: 48; TP: 44; AP: 32; AtmP: 48 and SP: 28. The maximum score for EC is 200. The data can be expressed as percentages of maximum scores in the respective subscale or of the global scale.

According to McAleer and Roff^29^ the items with an average value of≥3.50 are considered to be *"educational aspects of excellence"*; those between 3.01 and 3.49 are considered to be *"positive educational aspects"*; those with average values between 2.01 and 3.00 are considered to be *"educational aspects that could be improved"*; those ≤ 2.00 are defined as *"educational problematic areas"*, which should be examined more exhaustively later. In our study, we have considered the items with an average value >3 as positive aspects, those between 2.01 and 3 as educational aspects that could improve and those ≤ 2.00, educational problematic areas. The scores for global scale and subscales are grouped in 4 ordinal categories associated with a specific interpretation according to McAleer and Roff.^29^

We used a Spanish-language version of DREEM that has been validated and used previously.^9^^-^^10^^, ^^21^^-^^23^^, ^^25^^, ^^27^^-^^28^ However, this version and the original English version8 were revised by a group of Spanish medical educators, belonging to different medical schools, in order to correct some of the idiomatic differences that exist between the Spanish spoken in South American countries and Spain. The final questionnaire was applied to all participating medical schools. Prior to administration, a small pilot group of students from the University of Barcelona Medical School answered the questionnaire to ensure that the different items were well understood.

A short demographic questionnaire was constructed to collect information such as the participant's gender, age group, and course (2nd or 4th year).

### Sample size and data collection

The total students registered in the 2nd and 4th years and therefore potential respondents to the survey, were 2,049 (1,051 2nd year and 998 4th year respectively). The DREEM questionnaire was delivered directly in the classroom to the students at the end of the academic year (May 2013 and 2014). The permission to collect data was given by the Dean's office and students from each medical school.

Before distribution of the questionnaire, each collaborator explained to the participating students the objectives of the study and how the data would be processed, placing a special emphasis on the importance of voluntary participation and the anonymity of the process. Information on age, gender and academic year was collected from each participant. None of the information collected was identifiable, thereby maintaining data anonymity.

### Data analysis

The variables were described using means and standard deviations (SD) and percentages of maximum scores. Data were analyzed using SPSS16 software. Analysis of the data included comparisons of the DREEM mean global and subscale scores between male and female students, and between different years.  Different medical schools were not compared directly. Continuous variables were summarized as means and an independent t-test was used to determine differences in the scores corresponding to the five domains between the two courses (2nd and 4th years) in each medical school. A two-tailed Student's t-test was adopted to determine statistical significance of observed differences. Values of p<0.05 were considered significant. Examination of the reliability was limited to an analysis of the internal consistency by means of Cronbach's α.

## Results

### Participant demographics

Table 1 shows the number and percentage of participants by gender, age group, course and medical school.

From a total of 2,049 students, 1,513 medical students (74%) from the five participating medical schools answered the DREEM questionnaire. Of these, 894 students (59.8%) were in their 2nd year and 619 students (40.2%) were in the 4th year of the new curriculum. The response rate for the different universities ranged from 90% to 74% with an average of 85% in the 2nd year and from 69% to 50% with an average of 62% in the 4th year. The average ages were 19.5 and 21.8 for the 2nd and 4th year students, respectively. In the 2nd year, 30.68% of students were male and 69.32% female. In the 4th year, 28.7% students were male and 71.3% female.

**Table 1:** Student's response rate

Medical School	2^nd^year*	Frequency%	4^th^year*	Frequency%
Barcelona	221 (85%)	17.94	129 (50%)	20.84
Internacional de Catalunya	72 (90%)	5.85	48 (60%)	7.75
Las Palmas de Gran Canaria	131 (87%)	10.64	59 (54%)	9.53
Santiago de Compostela	297 (84%)	24.1	241 (69%)	38.93
Zaragoza	173 (74%)	14.05	142 (51%)	22.95
Total	894 (85%)	100	619 (62%)	100
Average age	19.5±2.4		21.8±1.8	
Male	274	30.68%	178	28.7%
Female	620	69.32%	441	71.3%

*Percentage of respondents are shown in parentheses versus total students in each year and university

### Analysis of reliability of DREEM questionnaire

The Cronbach's α coefficients of the questionnaire applied to 2nd year and 4th year students were 0.92 and 0.90 respectively. When the Cronbach's α coefficients for each subscale was considered separately, the values for 2nd and 4th year students ranged from 0.85 to 0.47 and from 0.80 to 0.56, respectively. In all cases, the lowest values were observed in the social perception subscale.

### EC global perception by medical students from the 2 and 4 years

The EC global scale average score for all 2nd year students from the five medical schools involved in this study (n=894) was 116.25±24.9 (58.2% of maximum value) reflecting an educational climate more positive than negative. The mean scores in the five different subscales were: LP: 25.5±7.3 (53.1%, a more positive perception); TP: 26.0±6.9 (59.0%, moving in the right direction); AP: 19.8±4.9 (61.8%, feeling more on the positive side); AtmP: 30.9±5.9 (64.3%, a more positive atmosphere); SP: 16.3±4.0 (58.2%, not too bad). The differences that were observed between males and females in the global scale and in the subscales were found to be non-significant (Table 2).

The EC global scale average score for all 4th year students (n=619) was 104.8±29.5 (52.4%) also reflecting *"an educational climate more positive than negative"*. However, this score was significantly lower with respect to the aggregated score of the 2nd year students considered. The score is at the threshold of the lower category considered as *"plenty of problems"* (51-100), according to McAleer and Roff.^2^ The mean scores in the different subscales were: LP: 21.4±9.0 (44.5%, teaching is viewed negatively); TP: 24.9±7.5 (56.5%, moving in the right direction); AP: 18.0±5.5 (56.2%, feeling more on the positive side); AtmP: 26.0±7.9 (54.1%, a more positive atmosphere); SP: 13.9 ±4.3 (49.6%, not a nice place). As was the case for 2nd year students, no significant gender-based differences were observed in the global scale or in the subscales (Table 2).

**Table 2:** Scores in global EC scale and subscales for 2nd and 4th year students

Global ECscales and subscales	2^nd^ year students(n=894)		4^th^ year students(n=619)	
	Mean ± SD	Maximum possible score	Mean ± SD	Maximum possible score
Global EC (200)	116.2 ± 24.9	58.2%	104.8 ± 29.5	52.4%***
LP (48)	25.5 ± 7.3	53.1%	21.4 ± 9.0	44.5%***
TP (44)	26.0 ± 6.9	59.0%	24.9 ± 7.5	56.5%
AP (32)	19.8 ± 4.9	61.8%	18.0 ± 5.5	56.2%
AtmP (48)	30.9 ± 6.9	64.3%	26.0 ± 7.9	54.1%**
SP (28)	16.3 ± 4.0	58.2%	13.9 ± 4.3	49.6%**

**p < 0.001 with respect to 2nd year students; ***p < 0.0001 with respect to 2nd year students

### Individualised EC perception in each medical school

#### 2nd year students (n= 894)

The individualized results of the different schools are shown in Table 3. Regarding the global scale, all medical obtained scores above 101 *("More positive than negative EC"*). In the LP subscale, two medical schools, (M2 and M5 showed scores < 25 indicating *"Teaching is viewed negatively"*. In the TP subscale one medical school gave a score below <23 (M5 indicating *"A need for some retraining*". In the AP subscale all medical schools obtained scores above 17 indicating that students *"Feel more on the positive side"*. In the AtmP subscale, all medical schools showed a score higher than 25 indicating *"A more positive atmosphere"*. Finally, in the SP subscale all medical schools obtained scores higher than 15, indicating that it is *"A place that is not too bad".*

When each medical school was considered separately, the percentage of students with scores lower than 100 ranged between 5.5% and 48%.  The percentage of students with scores in the 101-150 range varied between 42.0% and 73.3%. Finally, the percentage of students with scores higher than 150 ranged from 1.02% to 21.7% (Table 4).

**Table 3:** Comparative results between the 2nd and 4th years

Medical School	Subscale	2^nd^YearMean ±SD	% of maximum possible score in the global scale and subscales	4^th ^YearMean ±SD	% of maximum possible score in the global scale and subscales	*p-value*
M1	Learning	27.6±6.6	57.5	24.3±7.2	50.5	**
	Teacher	28.1±6.0	62.8	27.7±6.4	62.9	NS
	Academic	20.2±4.5	63.1	20.5±4.5	64.0	NS
	Atmosphere	32.1±6.6	66.8	30.2±6.2	62.9	NS
	Social	16.4±6.6	58.5	15.4±6.2	55.0	NS
	Global scale	124.5±23.1	62.2	116.8±20.0	58.4	**
M2	Learning	24.5±7.0	51.0	13.4±6.0	27.0	***
	Teacher	25.9 ±5.9	58.8	19.7±7.0	44.7	***
	Academic	19.6±4.7	60.3	13.9±4.0	43.4	***
	Atmosphere	29.5±7.3	61.4	18.6±12.0	38.7	***
	Social	15.1±4.0	53.9	11.8±3.9	42.1	***
	Global scale	114.8±24.2	57.4	79.1±21.3	39.5	***
M3	Learning	32.6±5.7	67.9	30.0±7.2	62.5	NS
	Teacher	29.1±6.2	66.1	29.5±6.8	67	NS
	Academic	21.9±4.5	68.4	20.9±5.6	65.3	NS
	Atmosphere	31.8±6.3	66.2	30.6±6.8	63.7	NS
	Social	18.4±3.6	65.7	16.1±4.3	57.5	NS
	Global scale	133.9±22.7	66.5	127.3±27.2	63.6	NS
M4	Learning	25.4±7.2	52.9	21.4±7.2	44.5	***
	Teacher	26.4±4.5	60.0	26.4±5.6	60.0	NS
	Academic	18.3±5.2	57.1	16.6±5.3	51.8	**
	Atmosphere	29.3±5.9	61.0	25.3±7.1	52.7	***
	Social	15.7±4.0	56.0	12.8±4.0	45.7	***
	Global scale	115.8±22.5	57.9	102.7±24.1	51.5	**
M5	Learning	24.9±5.5	51.8	21.8±6.5	45.4	***
	Teacher	22.6±4.5	51.3	25.1±5.1	57.0	NS
	Academic	19.7±3.8	61.5	19.1±4.4	59.6	NS
	Atmosphere	27.3±5.1	56.2	27.1±5.7	56.4	NS
	Social	16.6±3.4	59.2	16.6±3.4	59.2	NS
	Global scale	113.12±17.5	56.5	109.8±20.1	54.9	**
Mean of all Medical Schools	Learning	25.1±7.6	52.9	21.4±9.0	44.5	**
	Teacher	25.3±6.2	57.5	24.9±7.5	56.5	NS
	Academic	19.3±5.0	60.3	18.0±5.5	56.2	NS
	Atmosphere	29.0±7.2	60.4	26.0±7.9	54.1	**
	Social	15.9±4.1	56.7	13.9±4.3	49.6	**
	Global scale	115.6±25.6	57.8	104.8±29.5	52.4	***

**p < 0.001; ***p < 0.0001

**Table 4:** Percentage of students in the different score ranges in each medical school

Medical School	Scores <100% very poor or plenty of problems		Scores 101-150% more positive than negative		Scores >150% excellent	
	2^nd^	4^th^	2^nd^	4^th^	2^nd^	4^th^
M1	48.4	30.2***	42.0	64.3***	9.6	5.5***
M2	23.2	78.8***	68.2	21.2***	8.6	0***
M3	5.5	18.7***	72.8	58.3***	21.7	23.0
M4	24.4	42.3***	70.2	57.7***	5.4	0***
M5	23.2	25.6	73.3	74.02	1.02	0.38
Mean Medical Schools	26.6	40.0***	66.36	56.96***	7.04	3.4***

***p < 0.0001 with respect to the other groups

#### 4th year students (5 medical schools; n= 619)

The results obtained from 4th year students of the different schools are shown in Table 3. With one exception (M2), all medical schools obtained scores higher than 101 on the global scale indicating *"An EC More positive than negative*". In the LP subscale, four medical schools (M1, M2, M4 and M5) showed scores<25 indicating, *"Teaching is viewed negatively"*. In the TP subscale only one medical school (M2) showed a score < 23 indicating a *"Need of some retraining"*. In the AP subscale three medical schools (M1, M3 and M5) obtained scores > 17 indicating that students *"Feel more on the positive side"*. In the AtmP subscale, only one medical school (M2) showed a score < 25 showing that *"There are many issues, which need changing"*. Finally in the SP subscale, two medical schools (M2 and M4 obtained scores < 15 indicating that *"They are not a nice place"*.

With regards to the different medical schools, the percentage of students with scores lower than 100 ranged between 18.7% and 78.8%. The percentage of students with scores ranging between 101 and 150 varied between 21.2% and 74.02%. Finally, the percentage of students with scores higher than 150 ranged from 0% and 23.0% (Table 4).

### Comparing the results obtained in the two years (2 and 4)

We observed in M1, significant differences in the global scale and the LP subscale scores, with both being significantly lower in the 4th year than in the 2nd year. In M2 significantly lower scores were observed in the 4th year in the global scale and in all the subscales. In M3 no significant differences were observed either in the global scale or in subscales. In M4 significantly lower scores were observed in the 4th year, in the global scale and in all subscales, with the exception of the TP subscale. Finally, in M5 significantly lower scores were observed in the 4th year, in the global scale and in the LP subscale.

### Analysis of subscale items: a comparative analysis between 2nd and 4th year students in all medical schools (Table 5)

#### Students' Perceptions of Learning

In the 2nd year, 2 of the 12 items in this subscale scored < 2 and the rest scored between 2 and 3. In the 4th year, only 9 items scored > 2, indicating that there are many problematic areas in the learning domain where a general improvement is needed.

####  Students' Perceptions of Teachers

In both years, one item, *("The teachers are knowledgeable"*), from the eleven items of this subscale, scored > 3. In the 2nd year all of the other items scored between 2 and 3 indicating that aspects of this domain could be improved. However, in the 4th year one item scored < 2 *("The teachers are good at providing feedback to students"*), indicating a problematic area.

#### Students' Academic Self-Perception

In the 2nd year, all eight items in this subscale gave scores between 2 and 3, indicating areas that need to be improved. In the 4th year, the results were the same except for item 21 *("I feel I am being well prepared for my profession"*) which scored < 2 indicating a problematic area.

#### Students' Perceptions of Atmosphere

Only, one item of the twelve included in this subscale (item 33, *"I feel comfortable in the class socially")* scored > 3 in both the 2nd and 4th years. In both years, one item (item 12, *"The school is well timetabled")* scored < 2, indicating a problematic area. Another item (item 17, *"Cheating is a problem in this school"*) scored < 2 in the 4th year only, indicating another problematic area. Furthermore, in the 4th year item 43 *("The atmosphere motivates me as a learner"*) scored 2, a significantly lower value than in the 2nd year. The rest of the items scored between 2 and 3 indicating areas to improve. 

**Table 5:** Subscales analysis

Subscale: learning perception	Mean 5 schools2^nd^ year	Mean 5 schools4^th^year
1 I am encouraged to participate in class	2.1±0.98	1.4±0.18 **
7 The teaching is often stimulating	2.2±0.99	2.0±0.62
13 The teaching is student centred	2.1±1.08	1.3±0.32**
16 The teaching helps me to develop my competence	2.7±0.97	2.4±0.21
20 The teaching is well focused	2.2±1.10	1.7±0.57**
22 The teaching helps to develop my confidence	2.3±1.09	1.9±0.62**
24 The teaching time is put to good use	2.1±1.10	1.6±1.06**
25 The teaching over-emphasises factual knowledge	1.3±1.17	1.2±0.99
38 I am clear about the learning objectives of the course	2.5±1.00	2.3±0.74
44 The teaching encourages me to be an active learner	2.5±1.03	1.9±0.69**
47 Long term learning is emphasised over short term learning	1.9±1.10	1.6±1.07
48 The teaching is too teacher-centred	2.0±1.16	1.3±0.13**
Subscale: teaching perception		
2 The teachers are knowledgeable	3.1±0.90	3.1±0.10
6 The teachers are patient with patients	2.3±0.80	2.3±0.23
8 The teachers ridicule the students	2.6±1.22	2.4±0.39
9 The teachers are authoritarian	2.1±1.14	2.0±0.23
18 The teachers have good communication skills with patients	2.3±0.80	2.4±0.21
29 The teachers are good at providing feedback to students	2.0±0.95	1.6±0.47**
32 The teachers provide constructive criticism here	2.2±1.03	2.1±1.14
37 The teachers give clear examples	2.4±0.92	2.4±0.31
39 The teachers get angry in class	2.4±1.19	2.4±1.17
40 The teachers are well prepared for their classes	2.5±0.95	2.5±0.04
50 The students irritate the teachers	2.4±1.17	2.6±0.27
Subscale: academic perception		
5 Learning strategies which worked for me before continue to work for me now	2.1±1.27	2.1±0.04
10 I am confident about my passing this year	3.0±1.04	2.8±0.57
21 I feel I am being well prepared for my profession	2.5±1.00	1.8±0.56**
26 Last year's work has been a good preparation for this year's work	2.6±1.01	2.2±0.52
27 I am able to memorise all I need	2.1±1.11	2.0±0.73
31 I have learned a lot about empathy in my profession	2.4±1.13	2.4±0.45
41 My problem solving skills are being well developed here	2.3±1.01	2.2±0.71
45 Much of what I have to learn seems relevant to a career in healthcare	2.7±1.12	2.5±0.27
Subscale: atmosphere perception		
11 The atmosphere is relaxed during the ward teaching	2.5±0.90	2.3±0.27
12 The school is well timetabled	1.2±1.20	1.5±0.63
17 Cheating is a problem in this school	2.1±1.24	1.5±0.32
23 The atmosphere is relaxed during lectures	2.7±1.57	2.3±0.47
30 The teachers are good at providing feedback to students	2.4±1.01	2.1±0.01
33 I feel comfortable in class socially	3.2±1.02	3.2±0.65
34 The atmosphere is relaxed during seminars/tutorials	2.8±1.01	2.6±0.20
35 I find the experience disappointing	2.9±1.25	2.5±0.33
36 I am able to concentrate well	2.6±1.04	2.6±0.27
42 The enjoyments outweighs the stress of the course	2.4±1.19	2.1±0.72
43 The atmosphere motivates me as a learner	2.5±1.05	2.0±0.66**
49 I feel able to ask the questions I want	2.6±1.17	2.5±0.45
Subscale: social perception		
3 There is a good support system for students who get stressed	0.9±1.07	0.8±0.80
4 I am too tired to enjoy the course	1.9±1.27	1.4±0.23
14 I am rarely bored on this course	1.7±1.08	1.6±0.24
15 I have good friends in this school	3.4±1.01	3.5±0.34
19 My social life is good	2.8±1.17	2.4±0.15
28 I seldom feel lonely	2.8±1.25	2.6±0.31
46 My accommodation is pleasant	2.7±1.13	2.6±0.79

**p < 0.001

**Table 6:** Items scores of different medical schools in the 2nd year and in the 4th year

Items	2^nd^ Year					4^th^ Year				
	M1	M2	M3	M4	M5	M1	M2	M3	M4	M5
1	2.3	1.8	2.7	2.2	2.0	18	1.2	2.9	2.3	1.8
2	3.2	2.7	3.1	3.1	2.8	3.4	2.7	3.2	3.4	2.8
3	0.8	0.8	1.9	0.8	1.0	0.6	0.5	1.6	0.5	1.0
4	1.8	2.0	2.2	2.0	1.9	1.6	0.9	1.6	1.2	1.7
5	2.1	2.4	2.3	1.8	2.2	2.0	2.0	1.8	2.1	2.5
6	2.4	2.5	2.8	2.2	2.1	2.5	2.1	2.6	2.6	2.3
7	2.5	2.2	2.9	2.0	2.1	2.2	1.2	2.6	1.9	1.8
8	2.7	2.8	2.7	2.7	2.1	2.6	2.0	3.3	2.4	2.5
9	2.3	2.1	2.2	2.0	1.9	2.1	1.5	2.6	22	2.0
10	2.9	3.1	3.2	2.8	3.0	3.2	2.6	2.8	2.2	3.0
11	2.4	2.6	3.0	2.2	2.4	2.7	1.8	2.6	2.6	2.2
12	1.6	1.4	1.5	1.3	0.9	1.4	0.9	1.3	0.3	1.6
13	2.2	1.8	2.8	2.1	1.7	1.7	0.7	2.3	1.4	1.4
14	1.8	1.7	2.2	1.6	1.6	1.8	1.3	2.0	1.4	1.7
15	3.5	3.3	3.5	3.5	3.3	3.5	3.4	3.3	3.4	3.5
16	2.7	2.6	3.1	2.7	2.8	2.3	1.8	3.0	2.4	2.6
17	2.3	2.0	2.5	2.3	2.0	1.6	1.3	1.6	1.5	1.6
18	2.4	2.3	2.9	2.3	2.4	2.4	1.9	2.5	2.4	2.6
19	2.7	2.8	3.1	2.7	3.0	2.3	2.0	2.3	1.9	2.9
20	2.5	2.0	3.1	2.1	2.0	2.5	0.7	2.6	1.7	1.8
21	2.9	2.3	3.0	2.4	2.2	2.3	0.7	3.0	1.8	2.1
22	2.4	2.3	2.8	2.2	2.3	2.1	1.1	2.8	1.9	2.1
23	2.8	2.6	2.7	2.6	2.5	2.7	1.6	2.7	2.3	2.6
24	2.4	1.9	2.4	1.9	1.8	2.0	0.7	2.1	1.2	2.0
25	1.6	1.5	2.1	0.1	1.5	1.3	1.2	1.9	1.0	1.1
26	2.7	2.3	2.7	2.7	2.6	2.7	1.5	2.5	2.3	2.4
27	2.2	2.3	2.5	2.1	2.0	2.2	1.7	2.0	1.6	2.2
28	2.9	2.7	2.7	2.9	2.7	2.7	2.2	2.4	2.5	2.8
29	2.1	2.1	2.7	2.0	1.9	1.8	0.9	2.4	1.7	1.8
30	2.4	2.3	2.8	2.4	2.3	2.2	1.4	2.5	2.0	2.3
31	2.1	2.2	2.7	2.4	2.7	2.7	0.6	3.2	2.2	2.8
32	2.4	2.0	2.5	2.3	2.2	2.1	2.7	2.6	2.1	2.2
33	3.3	3.0	2.9	3.2	3.1	3.1	2.2	3.1	3.0	3.4
34	3.0	2.8	2.9	2.4	2.7	2.9	1.7	3.0	2.5	2.7
35	3.0	2.8	2.7	3.0	3.0	2.8	2.3	2.8	2.6	2.7
36	2.6	2.7	2.7	2.6	2.5	2.7	1.6	2.5	2.4	2.9
37	2.7	2.1	2.8	2.6	2.4	2.7	1.4	2.7	2.4	2.6
38	2.5	2.3	2.8	2.5	2.7	2.0	1.9	2.7	2.3	2.8
39	2.5	2.6	2.7	2.3	2.2	2.3	1.6	2.8	2.3	2.4
40	2.7	2.1	2.8	2.7	2.7	2.7	1.3	2.5	2.5	2.9
41	2.4	2.0	2.5	2.1	2.7	2.2	1.2	2.5	2.0	2.7
42	2.5	2.4	2.8	2.4	2.3	2.4	1.1	2.4	1.7	2.2
43	2.7	2.3	2.8	2.3	2.5	2.4	1.1	2.5	1.5	2.5
44	2.6	2.3	2.9	2.3	2.4	2.2	1.1	2.6	1.94	2.4
45	2.7	2.8	3.1	2.6	2.7	2.6	2.3	2.8	2.4	2.7
46	2.6	2.1	2.9	2.2	3.3	2.6	1.2	2.8	1.6	3.6
47	2.0	1.5	2.6	2.0	2.3	1.6	0.7	2.2	1.5	2.1
48	2.1	1.9	2.4	2.1	1.9	2.0	1.2	2.4	1.6	1.9
49	2.9	2.5	2.9	2.8	2.1	2.7	1.9	2.57	2.9	2.6
50	2,5	2,5	2,5	2,3	2.4	2.5	1.9	2.5	2.3	2.8

#### Students' Social Self-Perception

This subscale included seven items. The results were the same in both years. Three items (items 3, 4 and 14) ("There is a good support system for students who get stressed"; "I am too tired to enjoy the course" and "I am rarely bored on this course") scored below 2 indicating problematic areas. One item, (15: "I have good friends in this school") scored above 3. The remaining three items scored between 2 and 3 indicating areas to improve.

### Item analysis

When the different items of DREEM in all medical schools were analysed, the percentage of items scoring > 3 in the 2nd year is the same as in the 4th year (6%). The percentage of items scoring < 2 is significantly lower in the 2nd year compared to the 4th year (12% vs 32%). Finally, the percentage of items scoring between 2.00 and 3.00 was 82% in the 2nd year and 62% in the 4th year. In Table 6, the individualized results of the scores for the different items in each medical school for both years are shown.

## Discussion

EC is a key element of student learning reflecting the quality of the curriculum.^1^ Moreover, it has been considered the expression, the manifestation and the measure of a curriculum, as well as an expression and a stimulus for change.^1^^,^^7^ Since 2009, Spanish Medical Schools have been involved in a curricular reform to adapt to the Bologna Process. Taking this into account, SEDEM made a call for collaboration with different medical schools, in order to undertake a cross-sectional and comparative study to analyse the perception of EC by 2nd and 4th year students with the goal of detecting the strengths and weaknesses in this process, particularly during the transition from the basic to the clinic period.

To measure EC we decided to use the DREEM questionnaire because it was developed specifically to evaluate the EC in institutions of undergraduate medical education and has recently been recommended as the most suitable tool for this purpose.^30^ Moreover, DREEM has been used as an evaluation measure to diagnose deficiencies in the current EC, to compare the experiences of different groups within the EC, and to compare actual experiences of the EC with an ideal/expected for the same group. Given the reported difficulties in establishing meaningful comparisons across institutions to guide good practice^31^ and to avoid ranking the medical schools, our main focus was not on the comparison of different medical schools with each other. In our study we use the Spanish version of the DREEM questionnaire, which has been previously used and validated. ^9^^,^^10^^,^^25^

Only five of the forty medical schools in Spain (4 public and 1 private medical school) decided to participate in the study. This low participation perhaps suggests that the responsible authorities of many medical schools either do not give a high priority to obtaining information about the educational climate at their institution as perceived by its students or they do not give importance to these aspects as a determinant of the quality of the curriculum. We are convinced of the need for more schools to join future studies and SEDEM is ready to give its immediate support to any Spanish medical school that wants to enrol in this study, thus motivating them to develop a quality evaluation culture.

According to data taken from the current literature and the review of Miles^31^ we can affirm that the present study, including 1,513 medical students, represents one of the largest multicentre surveys carried out in medical schools and the first in Spain, in which EC has been evaluated using the DREEM scale. A similar study with 1,391 dentistry students was carried out comparing seven public Spanish dentistry schools.^2^^,^^5^ Other large-scale studies have been conducted in countries as Chile,^10^ (1,092 subjects) Germany,^19^ (1,119 subjects) and the UK,^14^ (968 subjects). Other studies have been carried out with smaller numbers of students. According to a recent publication^32^ our study is second in size only to the study reported at the Korean Medical Education Conference, which surveyed 9,096 students from 40 of the 41 medical schools in that country.

The percentage of respondents varied between medical schools, although in all cases the level of participation was always higher than 50%. It can be noted that participation levels were always higher in the 2nd year compared to the 4th year. The lower level of participation of 4th year students could partially explain the lower scores observed in this group, as discussed below. The results were expressed as mean values of the global scale, subscales or items and as a percentage of students in each ordinal category associated with a specific interpretation. An assessment of these percentages provides a different analytical focus on EC perceptions when compared to that obtained through simple comparisons of means or medians of the global scale, subscales or items.^13^

In our study, the observed Cronbach's α coefficient showed in general a high reliability in the global scale and the different subscales with the exception of the SP subscale. These data are in agreement with the results obtained in previous studies.^6^^,^^9^^,^^10^^,^^16^^,^^19^^-^^20^

Our study demonstrated that 2nd year medical students from all medical schools indicated that they generally perceived an EC more positive than negative (116.2±24.9). Likewise, 4th year students also perceived an EC that is more positive than negative. However, the score was significantly lower than in the 2nd year (104.8±29.5). When we analysed the percentage of students in each scoring category (<100, 101-150 and <150) we detected an important percentage of 2nd year students perceiving EC as very poor with plenty of problems (26.6%). This percentage was significantly larger for 4th year students (40%).  In general, these data indicate that improvements are needed in many areas.

These results agree with the majority of studies carried out in medical students that reported scores ranging between 101 and 140,^2^^,^^6^^,^^10^^,^^19^^,^^20^ but are lower than some other studies that showed scores above 141.^18^^,^^33^ Our results are consistent with those described by Roff et al.,^34^ who pointed out that medical schools with a traditional curriculum seem to score below 120. It is interesting to note that in our study the only medical school that scored>130 was the most recently founded (in 2004) of the five involved.

The overall results obtained for 2nd and 4th year students when aggregated together do not allow the identification of specific educational problems in each year, in each subscale or in a specific medical school. Thus we analysed the individualised results in the 2nd and 4th years in general, in each subscale, in each item, and in each medical school. The overall perception in all medical schools involved in this study is more positive than negative but the results obtained from the 4th year indicate that the perception is significantly lower than in the second year.  This observation, which again indicates a worsening of the EC in a clinical year, agrees with some studies^9^^,^^10^  but not with others.^19^

These lower results could be explained in part by the lower participation rate in the 4th year and also because the questionnaire was applied to 4th year students in their first year after implementation of the new curriculum, whilst for those students in the 2nd year the new curriculum was implemented two years ago. This is consistent with the studies of Roff etal.^8^ and McAleer et al.^35^ who considered that curriculum changes that are usually undertaken in order to improve the overall learning environment for students are often stressful for both students and faculty. When we analysed the scores in the LP subscale two medical schools in the 2nd year and four medical schools in the 4th year gave low scores indicating that the general perception is that teaching is viewed negatively. This negative perception could be explained by the traditional curriculum and the traditional methodologies, which are still used in the majority of Spanish medical schools. In the TP subscale the general consensus in all medical schools in both years was that the students felt that teachers are moving in the right direction. Generally speaking, our students consider that their teachers are knowledgeable but the main problem is that they still use traditional methods to teach and they participate very little in faculty development activities. In our medical schools the mechanisms for faculty development are poorly established and teachers, especially clinicians, are not very interested in such aspects. In the AP and AtmP subscales, our students in general feel more on the positive side although some differences were observed between particular medical schools. Finally, in the SP subscale the general student's perception was not too bad.

When we analysed the different items in order to identify problematic aspects, in the learning perception subscale in the 2nd year, we detected two items scoring < 2, items 25 (The teaching over-emphasises factual knowledge) and 47, (Long-term learning is emphasised over short-term learning). These low scores indicate that in our medical schools, with their very traditional teaching methods, the focus is largely on the transmission of knowledge rather than on the student, precluding active and problem-based learning. In the 4th year the results are significantly lower. The low scores observed (< 2) in nine items, including items 25 and 47, provide further evidence that the focus of learning is centred more on the teacher than on the students and that this situation is more pronounced in the clinical period. The learning strategies used are conceived with a teacher-centred emphasis, which provokes non-stimulating teaching and a lack of feed-back amongst other problems.

In the teaching perception subscale, there are no items scoring <2, except item 29 (The teachers are good at providing feedback to students) for 4th year students. In our opinion this is a problematic area that requires urgent improvement. This problem seems to be a commonly encountered one, especially in clinical years.^2^^,^^6^^,^^9^^,^^13^^-^^14^^,^^19^^,^^20^

In both years one item scored above 3 (The teachers are knowledgeable). These results emphasise the quality of the teachers in our medical schools regarding their theoretical knowledge. The problem is that in general traditional teaching methodologies are still used.

In the academic self-perception subscale all the items scored between 2 and 3 indicating several areas where improvement is required. The only exceptions were in the 4th year where items 21 (I feel I am being well prepared for my profession) and 27 (I am able to memorise all I need) scored < 2. The low score for item 21 agrees with the results observed in the Learning subscale where learning is viewed negatively. The idea that teaching is focused on providing a lot of information and factual knowledge rather than on long-term learning is reinforced by the low score for item 27 and is also consistent with the frequent complaints from medical students that they receive a lot of information that is sometimes irrelevant or unnecessary.

In the atmosphere perception subscale in both 2nd and 4th years, one item scored > 3: item 33 (I feel comfortable in class socially). However, in both years the students considered that the school is not well timetabled. This is a common problem in all Spanish medical schools where students spend a lot of time in the medical school attending different activities and have little time for learning or self-learning activities. Other negative areas identified in the 4th year were item 17 (Cheating is a problem in this school) and item 43 (The atmosphere motivates me as a learner). Cheating, a problem that is considered a demotivating factor for some students could be a consequence of the high degree of competitiveness in Spanish medical schools and the anxiety to obtain the good marks required for success in the national examination to accede to a position for postgraduate training. Another possible cause could be an inadequate fairness of the tests, something that also been described in the literature.^9^^,^^36^

In the social perception subscale, both 2nd and 4th year students scored three items<2 indicating important problematic areas. One of these is item 3 (There is a good support system for students who get stressed). This appears to be a common problem in medical schools.^9^^,^^14^^,^^36^^,^^37^ Although a tutorial system for students exists in Spanish medical schools, its principal objective is to provide assistance in the academic field. In general a formal support service is not offered. Some authors have suggested improvements to this educational aspect via a number of methods including a more structured and accessible personal tutoring system, peer tutoring, an approachable chaplaincy service, better access to school office staff and senior to junior student mentoring.^38^^,^^39^ On the other hand, Whittle et al.^14^ recommended an improvement in the dissemination of information about the university student support system (which is available throughout the course), because students often forget how to access these services.

The other two items that scored lower in both years were item 4 (I am too tired to enjoy the course) and item 14 (I am rarely bored on this course). Finally, in this subscale, item 15 scored > 3, (I have good friends in this school). In spite of this, the perception of this subscale in the 4th year is not adequate.

In general, taking in account all these results, we can deduce that in all medical schools (with one exception) the perception of the educational climate by 2nd year students is more positive than the perception of their colleagues from the 4th year, although common problematic areas exist. There are particular problems in the 4th year with regards to the learning perception subscale. These could be explained by different factors that are not mutually exclusive: firstly, the students become more critical as they advance in their studies. Secondly, the environment worsens during clinical teaching and, thirdly, our 4th year students are the first cohort to experience the new curriculum.

In conclusion, our results show that the general perception of EC in the Spanish medical schools involved in the study is more positive than negative although it is significantly more negative in the 4th year. In spite of this general perception students outline the existence of several *"educational problematic areas"* associated with the persistence of the traditional curriculum and teaching methodologies. These problems are more evident in the 4th year where the students consider that the EC has plenty of negative aspects associated in part with a teacher-centred conception of teaching that can explain the lack of feedback, non-stimulating teaching, a demotivating atmosphere and the perception of not being well prepared for the profession. This study must drive medical schools to make a serious reflection in order to implement the necessary changes to improve teaching. This is especially true for the clinical period. From SEDEM, we will continue to call for the rest of the medical schools in Spain to involve themselves in this project as a tool to increase curriculum quality.

### Acknowledgments

We give thanks to Dr Jonathan Giblin for assistance in the proofreading of the manuscript. We also gratefully acknowledge the participation of all the students involved in this study and thanks are also due to the Deans of the Medical Schools that participated in this study.

### Conflict of Interest

The authors declare that they have no conflict of interest.

## References

[1] Genn JM (2001). AMEE Medical Education Guide No. 23 (Part 1): Curriculum, environment, climate, quality and change in medical education-a unifying perspective.. Med Teach.

[2] Roff S, McAleer S (2001). What is educational climate?. Med Teach.

[3] Plucker JA (1998). The Relationship Between School Climate Conditions and Student Aspirations.. The Journal of Educational Research.

[4] Pimparyon, S. M Caleer, S. Pemba, S P (2000). Educational environment, student approaches to learning and academic achievement in a Thai nursing school.. Med Teach.

[5] Lizzio A, Wilson K, Simons R. University students' perceptions of the learning environment and academic outcomes: implications for theory and practice. Stud Higher Educ. 2002(1); 27:27-52.

[6] Mayya S, Roff S. Students' perceptions of educational environment: a comparison of academic achievers and under-achievers at Kasturba Medical College, India. Educ Health (Abingdon). 2004 Nov;17(3):280-91. 10.1080/13576280400002445 15848815

[7] Harden RM (2001). The learning environment and the curriculum.. Med Teach.

[8] Roff S, McAleer S, Harden RM, Al-Qahtani M, Ahmed AU, Deza H (1997). Development and validation of the Dundee Ready Education Environment Measure (DREEM).. Med Teach.

[9] Riquelme A, Oporto M, Oporto J, Méndez JI, Viviani P, Salech F, et al. measuring students' perceptions of the educational climate of the new curriculum at the Pontificia Universidad Católica de Chile: performance of the Spanish translation of the Dundee ready education environment measure (DREEM). Educ Health. 2009;22(1):112. 19953435

[10] Herrera C, Pacheco J, Rosso F, Cisterna C, Daniela A, Becker S (2010). Evaluation of the undergraduate educational environment in six medical schools in Chile.. Rev Med Chil.

[11] Till H (2004). Identifying the perceived weaknesses of a new curriculum by means of the Dundee Ready Education Environment Measure (DREEM) Inventory.. Med Teach.

[12] Varma R, Tiyagi E, Gupta JK (2005). Determining the quality of educational climate across multiple undergraduate teaching sites using the DREEM inventory.. BMC Med Educ.

[13] Miles S, Leinster SJ (2007). Medical students' perceptions of their educational environment: expected versus actual perceptions.. Med Educ.

[14] Whittle SR, Whelan B, Murdoch-Eaton DG (2007). DREEM and beyond; studies of the educational environment as a means for its enhancement.. Educ Health (Abingdon).

[15] Al-Ayed IH, Sheik SA (2008). Assessment of the educational environment at the College of Medicine of King Saud University, Riyadh.. East Mediterr Health J.

[16] Aghamolaei T, Fazel I (2010). Medical students' perceptions of the educational environment at an Iranian Medical Sciences University.. BMC Med Educ.

[17] Denz-Penhey H, Murdoch JC (2010). Is small beautiful? Student performance and perceptions of their experience at larger and smaller sites in rural and remote longitudinal integrated clerkships in the Rural Clinical School of Western Australia.. Rural Remote Health.

[18] Edgren G, Haffling AC, Jakobsson U, McAleer S, Danielsen N. Comparing the educational environment (as measured by DREEM) at two different stages of curriculum reform. Med Teach. 2010;32(6): e233-8. 10.3109/01421591003706282 20515368

[19] Rotthoff T, Ostapczuk MS, De Bruin J, Decking U, Schneider M, Ritz-Timme S. Assessing the learning environment of a faculty: psychometric validation of the German version of the Dundee Ready Education Environ-ment Measure with students and teachers. Med Teach 2011; 33(11): e624-36. 10.3109/0142159X.2011.610841 22022916

[20] Brown T, Williams B, Lynch M (2011). The Australian DREEM: evaluating student perceptions of academic learning environments within eight health science courses.. Int J Med Educ.

[21] Díaz-Véliz G, Mora S, Escanero JF. El instrumento DREEM. In: Escanero JF, editor. Estilos, enfoques y contexto de aprendizaje. Zaragoza (Spain): Prensas Universitarias de Zaragoza; 2011.

[22] Díaz-Véliz G, Mora S, Bianchi R, Gargiulo PA, Terán C, Gorena D, et al. Percepción de los estudiantes de medicina del ambiente educativo en una facultad con currículo tradicional (UCH-Chile) y otra con currículo basado en problemas (UNC-Argentina). Educ Med. 2011;14(1):27-34.

[23] Díaz-Véliz G, Mora S, Escanero-Marcen JF. Análisis del ambiente educacional tras la implantación del Plan de Bolonia en la Facultad de Medicina de la Universidad de Zaragoza. España. Comparación con la Facultad de Medicina de la Universidad de Chile. FEM. 2013;16(3):167-179.

[24] Zawawi AH, Elzubeir M (2012). Using DREEM to compare graduating students' perceptions of learning environments at medical schools adopting contrasting educational strategies.. Med Teach.

[25] Tomás I, Casares-De-Cal MA, Aneiros A, Abab M, Ceballos L, Gómez-Moreno G, et al. Psychometric validation of the Spanish version of the Dundee Ready Education Environment Measure applied to dental students. Eur J Dent Edu. 2014;18(3): 162-9. 10.1111/eje.12073 24330078

[26] Al-Naggar RA, Abdulghani M, Osman M T, Al-Kubaisy W, Daher AM, Nor Aripin KN, et al. The Malaysia DREEM: perceptions of medical students about the learning environment in a medical school in Malaysia. Adv Med Educ Pract. 2014;5:177-84. 10.2147/AMEP.S61805 PMC4061139 24959093

[27] Escanero JF, Mora S, Arce J, Bianchi R, Díaz-Véliz G, Gargiulo PA, et al. El alumnado. In: Escanero JF, editor. Estilos de aprendizaje y currículum: propuestas de mejora. Zaragoza (Spain): Prensas Universitarias de Zaragoza; 2009.

[28] Tomás I, Milla U, Casares MA, Abad M, Ceballos L, Gómez-Moreno G, Hidalgo JJ, et al. Analysis of the 'Educational Climate' in Spanish Public Schools of Dentistry using the Dundee Ready Education Environment Measure: a multicentre study. Eur J Dent Edu. 2013; 17(3): 159-68. 10.1111/eje.12025 23815693

[29] McAleer S, Roff S. A practical guide to using the Dundee Ready Education Environment Measure (DREEM). AMEE Medical Education Guide. 2001 (23): 29-33.

[30] Soemantri D, Herrera C, Riquelme A (2010). Measuring the educational environment in health professions studies: a systematic review.. Med Teach.

[31] Miles S, Swift L, Leinster SJ (2012). The Dundee Ready Education Environment Measure (DREEM): a review of its adoption and use.. Med Teach.

[32] Roff S, McAleer S (2014). Robust DREEM factor analysis.. Med Teach.

[33] McKendree J (2009). Can we create an equivalent educational experience on a two campus medical school?. Med Teach.

[34] Roff S (2005). The Dundee Ready Educational Environment Measure (DREEM)--a generic instrument for measuring students' perceptions of undergraduate health professions curricula.. Med Teach.

[35] McAleer S, Roff S, Harden RM, Qahtani MA, Ahmed AU, Deza H (1998). The Medical Education Environment Measure: a diagnostic tool.. Med Educ.

[36] Al-Hazimi A, Al-Hyiani A, Roff S (2004). Perceptions of the educational environment of the medical school in King Abdul Aziz University,saudi Arabia.. Med Teach.

[37] Roff S, McAleer S, Ifere OS, Bhattacharya S (2001). A global diagnostic tool for measuring educational environment: comparing Nigeria and Nepal.. Med Teach.

[38] Dunne F, McAleer S, Roff SA. Assessment of the undergraduate medical education environment in a large UK medical school. Health Educ J. 2006; 65(2): 149-158. DOI: 10.1177/001789690606500205.

[39] Avalos G, Freeman C, Dunne F (2007). Determining the quality of the medical educational environment at an Irish medical school using the DREEM inventory.. Ir Med J.

